# Primary Gastric Synovial Sarcoma: A Report of a Rare Case and Review of the Literature

**DOI:** 10.7759/cureus.94738

**Published:** 2025-10-16

**Authors:** Ilia Gotsadze, Manana Jikurashvili, Mariam Gendzekhadze, David Chechelashvili, Lado Kostava, Armaz Mariamidze, Sopiko Matcharashvili, Salome Tchokhuri

**Affiliations:** 1 Oncology, American Hospital Tbilisi, Tbilisi, GEO; 2 Pathology, Pathology Research Centre, Tbilisi, GEO; 3 Pathology, Pathology Laboratory, CSD-Georgia, Tbilisi, GEO; 4 Pathology, Pathology Research Center, Tbilisi, GEO

**Keywords:** gastric tumors, gastrointestinal tract, immunohistochemistry, soft tissue pathology, spindle cell neoplasms, synovial sarcoma

## Abstract

Synovial sarcoma is typically a soft tissue neoplasm, but gastrointestinal tract involvement is unusual. When occurring in the stomach, its appearance may overlap with more common spindle cell tumors such as gastrointestinal stromal tumors (GISTs). We describe the case of a 44-year-old woman who presented with anemia and abdominal pain. Radiologic imaging revealed a large gastric mass with extragastric extension, initially interpreted as a possible GIST. Subsequent histopathological examination demonstrated a monophasic spindle cell tumor, and the immunohistochemical profile was consistent with synovial sarcoma. Molecular analysis further supported the diagnosis. This report emphasizes the diagnostic challenges of gastric synovial sarcoma and highlights the critical role of combining histopathology, immunohistochemistry, and molecular testing to achieve an accurate classification. Awareness of this rare entity is essential for timely diagnosis and optimal management.

## Introduction

Synovial sarcoma is a spindle cell tumor with varying extents of epithelial differentiation, including gland formation, and is genetically distinct due to specific chromosomal translocations. Although it is named synovial sarcoma, it does not originate from synovial tissue and can occur in regions unrelated to synovial structures, including the head and neck, thoracic cavity, and abdominal wall. Intra-abdominal and gastrointestinal presentations are particularly rare, with the stomach being an exceptional primary site [1].

Clinically, synovial sarcoma typically presents as a slow-growing mass, with or without pain [2,3]. Nearly all cases harbor the characteristic chromosomal translocation t(X;18)(p11.2;q11.2) [4,5]. Molecular confirmation using fluorescence in situ hybridization (FISH) or reverse transcriptase-polymerase chain reaction (RT-PCR) to detect the SS18::SSX fusion gene is the diagnostic gold standard, especially in challenging histologic cases [4,5]. With 100% of biphasic and 96% of monophasic cases carrying this translocation, SS18::SSX fusion has become a hallmark in diagnosing synovial sarcoma [4]. The translocation involves fusion of SS18 with SSX1, SSX2, or SSX4, and some evidence suggests that the SS18::SSX1 subtype may be associated with a worse prognosis compared to SS18::SSX2 [4].

Epidemiological studies indicate that synovial sarcoma occurs more frequently in certain ethnic groups, with a higher incidence reported in the Hispanic population compared to the non-Hispanic White population [6]. First-born status and higher birth weight have also been proposed as risk factors [6]. However, most cases are sporadic, with no consistent environmental or hereditary predispositions identified [4,6]. Visceral presentations remain particularly rare, largely described in isolated case reports, and no specific risk factors have been established for gastric synovial sarcoma [7-10].

Several retrospective studies have also evaluated the role of radiation therapy in retroperitoneal and non-lipomatous sarcomas, highlighting its potential importance in the management of rare visceral presentations [11-14].

## Case presentation

A 44-year-old woman presented with general weakness, dyspeptic symptoms, and abdominal pain. The patient's chronic conditions included hypothyroidism. In the previous month, she had initially presented to her family physician with generalized weakness, somnolence, and pallor due to acute anemia. As her condition did not improve with standard anemia treatment, the physician ordered additional diagnostic investigations, which subsequently raised the suspicion of an underlying neoplasm. On admission, blood tests confirmed severe anemia.

**Figure 1:**
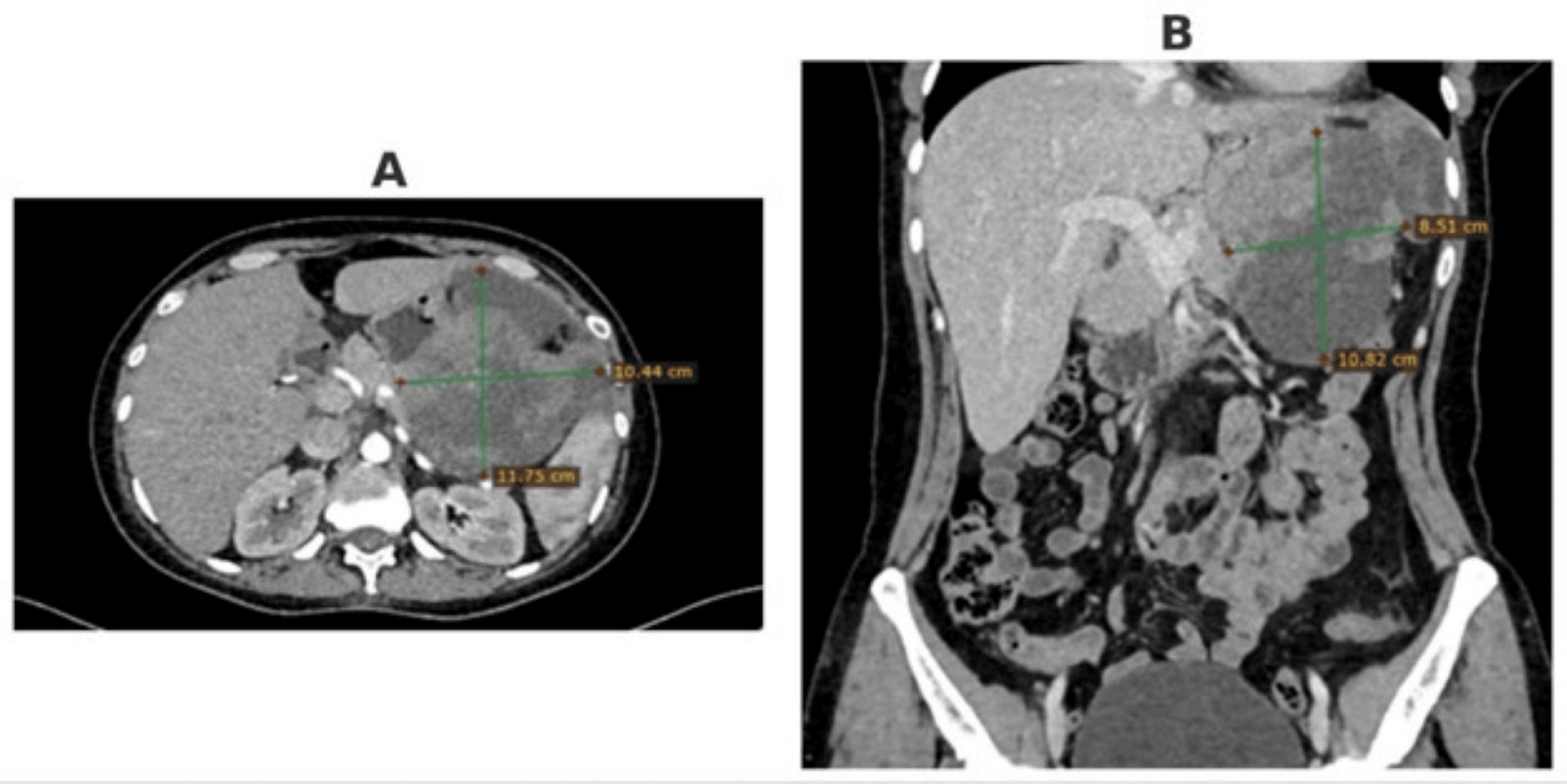
Computed tomography of the abdomen with intravenous contrast. (A) Axial view, (B) Coronal view.

An abdominal computed tomography (CT) scan with intravenous contrast demonstrated thickening of the stomach walls at the level of the fundus and greater curvature, with an irregularly shaped, fluid-tissue-density mass exhibiting extragastric spread, measuring 9.2 × 12.5 cm transversely and 13.2 cm craniocaudally. The mass had broad contact with adjacent viscera, including liver, pancreas, spleen, and major vessels, without clear fat planes, suggesting locally aggressive behavior. No evidence of distant metastases was noted. Given the resectable nature of the lesion, surgical management was planned (Figure 1).

The abdominal cavity was entered via a midline laparotomy. Initial exploration revealed a large tumor mass in the right upper quadrant of the abdomen, invading the anterior abdominal wall, the greater curvature of the stomach, the splenic hilum, and the left hemidiaphragm. There was also dense tumor infiltration involving the body of the pancreas and splenic vessels. A small amount of ascitic fluid was present.

**Figure 2:**
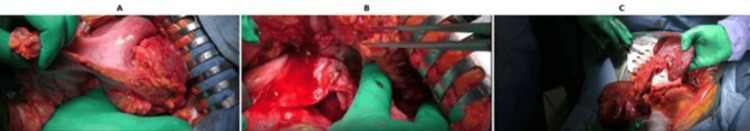
(A) Intraoperative view of the tumor following division of the gastrocolic ligament; (B) Tumor involving the pancreatic parietal peritoneum; (C) Final gross appearance of the resected specimen.

The procedure began with the separation of the tumor from the anterior abdominal wall. The gastrocolic ligament was then divided, and the transverse colon with its mesentery was mobilized away from the tumor. The splenic flexure of the colon was also mobilized. Division of the gastrosplenic ligament exposed direct tumor invasion of the splenic hilum. A decision was made to proceed with an en bloc splenectomy. The splenic vessels were divided at the hilum, and the spleen was mobilized from its ligamentous attachments and incorporated into the specimen (Figure 2).

The body of the pancreas and splenic vessels were then dissected free from the specimen. A circumferential resection of the diaphragm was performed, and the portion of the diaphragm infiltrated by the tumor was mobilized en bloc. Entry into the left pleural cavity was noted. The tumor was mobilized circumferentially and was attached only to the greater curvature of the stomach. The greater curvature was mobilized, and the stomach was transected atypically with a stapling device within macroscopically healthy margins. The specimen was removed en bloc (Figure 2).

**Figure 3:**
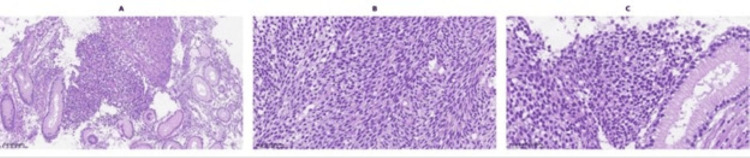
(A) Low-power view of the tumor showing a cellular proliferation composed of spindle-shaped cells arranged in fascicles, infiltrating between colonic glands (H&E, ×16); (B) Closer magnification demonstrating nuclear atypia and mitotic activity in the spindle-cell component (H&E, ×40); (C) Higher-power view highlighting the uniform spindle cells with elongated, hyperchromatic nuclei and scant cytoplasm, arranged in intersecting fascicles (H&E, ×40)

**Figure 4:**
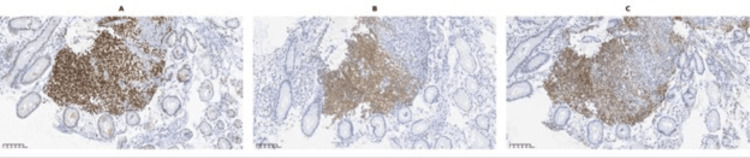
(A) TLE1 immunohistochemistry showing diffuse nuclear positivity in tumor cells (×20); (B) BCL2 immunohistochemistry demonstrating diffuse positivity in tumor cells (×20); (C) CD99 immunohistochemistry demonstrating diffuse positivity in tumor cells (×20).

**Figure 5:**
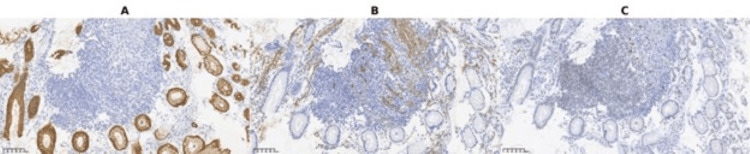
(A) EMA immunohistochemistry: tumor cells are negative, with preserved strong cytoplasmic staining in adjacent colonic epithelium (internal control) (×20); (B) SMA immunohistochemistry: tumor cells are negative, with positivity restricted to stromal smooth muscle bundles (internal control) (×20); (C) CD117 immunohistochemistry: tumor cells are negative, with scattered non-neoplastic elements showing weak staining (internal background) (×20). EMA: epithelial membrane antigen; SMA: smooth muscle actin

Histologically, the tumor was composed of spindle cells with elongated, hyperchromatic nuclei, monophasic features, and moderate mitotic activity (Figure 3). Immunohistochemical analysis demonstrated strong positivity for CD99, TLE1, Bcl-2, CD56, and epithelial membrane antigen (EMA), and negativity for CD117, DOG1, CD34, S100, smooth muscle actin (SMA), AE1/AE3, desmin, ALK, synaptophysin, CD45, and CD20. The immunoprofile, particularly TLE1 positivity combined with the absence of GIST markers (CD117, DOG1) and muscle markers, favored synovial sarcoma over other spindle cell tumors (Figures 4-5). A recommendation was made for molecular confirmation of the SS18 rearrangement.

## Discussion

Synovial sarcoma is rarely diagnosed primarily in the gastrointestinal tract, and its occurrence in the stomach poses diagnostic dilemmas [5]. Accurate diagnosis requires a thorough histopathological evaluation due to morphologic overlap with several histologic mimics, including gastrointestinal stromal tumors (GISTs), leiomyosarcomas, malignant peripheral nerve sheath tumors, and other sarcomas [4,5]. In our case, the absence of CD117 and DOG1 effectively ruled out GIST [3], while the lack of S100 and SOX10 expression argued against malignant peripheral nerve sheath tumor, and negative SMA/desmin excluded smooth muscle tumors [4].

The monophasic variant is particularly difficult to identify due to the absence of epithelial components. Therefore, immunohistochemistry plays a pivotal role [3,4]. In this case, positive TLE1, CD99, and Bcl-2 provided supportive evidence for the definitive diagnosis. However, TLE1 is not entirely specific, as it can be positive in other sarcomas, reinforcing the need for confirmatory molecular testing [4,8].

From a clinical perspective, primary gastric synovial sarcoma has no pathognomonic imaging features. Symptoms such as anemia and abdominal pain are usually related to mass effect or mucosal ulceration [5]. Treatment generally follows soft tissue sarcoma protocols: complete surgical excision with negative margins remains the mainstay [8]. Although several retrospective studies, including a large cohort of synovial sarcoma patients, have evaluated chemotherapy's impact on outcomes post-R0 resection, no significant improvement in overall survival (OS) or relapse-free survival (RFS) has been consistently observed, regardless of neoadjuvant or adjuvant use [1].

The role of radiation therapy in retroperitoneal sarcoma remains incompletely defined. Some of the large retrospective studies and registry analyses suggest that neoadjuvant radiation may be associated with improved local control and, in some analyses, improved OS, particularly in non-lipomatous histologies and larger tumors. Preoperative radiation may be considered in carefully selected patients, especially those with high-risk or bulky tumors, for the purpose of enhancing local disease control. But the evidence is mixed, and multidisciplinary discussion is essential for every case [10-12]. Long-term follow-up is essential due to the high risk of local recurrence and distant metastasis, particularly to the lungs and lymph nodes [13].

Prognosis for visceral synovial sarcoma is not well defined due to its rarity; however, tumor size greater than 5 cm, high mitotic activity, and incomplete resection are recognized as adverse prognostic factors [3,8]. Our patient’s large tumor size would be considered high-risk in most sarcoma grading systems, underscoring the importance of close surveillance.

## Conclusions

Synovial sarcoma of the stomach represents an exceptionally rare diagnostic entity, and awareness of this possibility is essential for both pathologists and clinicians. Because its histologic features can overlap with other spindle cell tumors, particularly GISTs, accurate diagnosis requires careful integration of morphology, immunohistochemistry, and molecular testing for the SS18::SSX fusion. A misdiagnosis can lead to suboptimal treatment, underscoring the importance of a multidisciplinary approach.

Complete surgical resection remains the cornerstone of management, as it offers the best chance for long-term survival. The role of adjuvant therapies such as radiotherapy and chemotherapy is less clearly defined in visceral presentations but may be considered in high-risk or incompletely resected cases. Early recognition of synovial sarcoma is therefore critical to guide appropriate surgical planning and ensure optimal oncologic outcomes. Despite advances in diagnostic and therapeutic modalities, prognosis remains guarded, especially in patients with large tumor size, high-grade histology, or adverse molecular features. Continued reporting of such rare cases adds to the collective understanding of gastric synovial sarcoma and may eventually contribute to the development of tailored management strategies in the future.
